# Importation and Transmission of Parasitic and Other Infectious Diseases Associated with International Adoptees and Refugees Immigrating into the United States of America

**DOI:** 10.1155/2015/763715

**Published:** 2015-10-25

**Authors:** Jordan Smith Darr, David Bruce Conn

**Affiliations:** ^1^One Health Center and Department of Biology, Berry College, Mount Berry, GA 30149, USA; ^2^Department of Invertebrate Zoology, Museum of Comparative Zoology, Harvard University, 26 Oxford Street, Cambridge, MA 02138, USA

## Abstract

Each year, hundreds of millions of people travel across international borders or even oceans, and up to 230 million may remain for long periods. Among these, 3–5 million settle permanently in their new homes, with about 1 million migrating permanently to the United States of America. This may result in transport of parasites and other pathogens, which might become established, infecting individuals in the new location. Beyond concern of disease spread, the health of migrants is of concern since the rigors, circumstances, and living conditions surrounding migrations may increase the vulnerability of migrants to infections. International adoptees and refugees are a small subset of these migrants but are of special significance inasmuch as adoptees may be more vulnerable to infection due to their immature immune status, and refugees may be more vulnerable due to substandard living conditions. Both originate from diverse regions, but often from environments of low hygiene and health care standards. This review examines recent examples of infections reported from adoptees and refugees entering the USA through 2010, highlighting the most common origin countries and the diseases most frequently involved, including Chagas disease, *Balamuthia* amebic meningoencephalitis, giardiasis, microsporidiosis, hepatitis, measles, pertussis, tuberculosis, malaria, intestinal helminths, and syphilis.

## 1. Background

In the past few decades, international adoptions and refugee cases have become increasingly common within the United States of America (USA or US). Between 1989 and 2000, US citizens adopted approximately 18,846 children from China alone, and the annual increase in adoptions is significant [1]. For example, US citizens adopted 20,099 children from 20 countries in 2002 compared with only 7,093 international adoption cases in 1990 [2]. This increase in permanent relocation between countries and cultures introduces a series of significant concerns for the health of both the immigrants and the US public at large. While this appears to be a general trend, however, it is important to note that levels of both international adoption and admission of international refugees are not always positive and fluctuate in response to national and international pressures. In this review, we do not attempt to provide a comprehensive analysis of US immigration policies regarding refugees and adoptees. This report is intended to examine the general issues, highlighting some representative recent examples from specific localities.

It is estimated that approximately 2% of the world's population resides in a nation other than the one in which they were born [3]. Although globalization has several positive consequences, it has also become a great concern as increased technology and economic means have afforded larger populations with access to intercontinental travel, and along with it the capacity to spread diseases rapidly on a global scale [4]. The resulting displaced, foreign-born population composed of immigrants, refugees, and adoptees not only experiences a shift in their personal health experience, but also impacts the health environments of the new communities into which they come to reside. Two of the most important factors directly relating to the epidemiology of disease due to migrations are the “degree of difference between origin and the destination” and the “size of the mobile population that moves between the different disease prevalence patterns” [4]. Although they comprise relatively small populations, foreign-born adopted children and international refugees may potentially play a significant role in the globalization of some infectious diseases. Not only have their numbers grown considerably in size in the past few decades, but they also represent populations migrating to considerable distances across diverse epidemiological regions, thus making them of increased relevance in the study of infectious disease.

However, it is important to note that the well-being of these legally accepted migrant populations is highly regulated and monitored in the USA, such that data comparing their infections with those of other migrant populations are not readily available at present. These populations are often overrepresented in epidemiological studies of the foreign-born migrants, simply because the administrative and legal requirements of their status generate data. The larger and nationally more significant foreign-born populations (including lawful permanent residents as well as undocumented/unlawful residents) are almost certain to have greater epidemiological impact. Nevertheless, while adoptees and refugees make up a relatively small component of overall US permanent immigrants, to ensure optimal health for them as well as their new neighbors, there is a need for vigilance regarding the pathogens and parasites associated with their migrations.

The United States Centers for Disease Control and Prevention (CDC) defines new and reemerging infectious diseases as “diseases of infectious origin whose incidence in humans has increased within the past two decades or threatens to increase in the near future” [5]. The emergence of an infectious disease is dependent upon the introduction of a disease-causing agent into a new population, reinforced by the disease's establishment and proliferation into the new region [6]. While developed nations have achieved complete or relatively high success in controlling most infectious disease transmission, underdeveloped nations are still struggling with the means to control and treat such diseases, or to provide preventative care. Most developed countries do not routinely perform screening for many nonendemic infections potentially arriving from abroad, so that the probability of spreading of such diseases increases. Foreign children awaiting adoption by parents in another country are often exposed to a variety of infectious diseases due to inferior standards for immunization practices and lack of preventative care within the health care systems of some underdeveloped countries. Within the close confines of an orphanage, transmission of infectious diseases including tuberculosis, hepatitis B, measles, intestinal parasites, bacterial pathogens, and various viruses has been observed between children and caretakers. Studies have shown that “infants and young children who are brought together in groups for care have a higher rate of infection, greater severity of illness, and increased risk for acquisition of resistant organisms” [7]. Furthermore, these same diseases, often untreated before departure from the originating country, are brought into the homes of adoptive parents and caretakers, who thus experience increased risk of exposure to foreign pathogens, potentially leading to new or reemerging cases of infectious disease.

As with international adoptees, international refugees have come to form a relatively small, yet significant, population within the United States. Refugees can be defined as “individuals who are outside their country and cannot return because of a well-founded fear of persecution related to their race, religion, and political or social affiliations” [8]. Like the foreign-born adopted child population, the number of international refugees has also greatly increased in recent decades. In the 1960s, the world refugee population was approximately 1 million, and yet in 2003 it had rapidly grown to include 11.9 million people [9]. The conditions to which this unique population is exposed before departure, during transit, and after arrival in destination camps afford them little or no health care and an increased risk of infectious disease. Between 1979 and 2004, 75,000 refugees settled in the US state of Minnesota alone, and thousands more established residence throughout the United States. All of this has the potential to impact the epidemiology of infectious diseases in the communities in which they have newly settled [9]. The growing refugee population, combined with frequently unsanitary conditions and inadequate hygiene, makes this a group of particular concern.

This review serves to highlight the findings of numerous case studies and reviews in this arena of public health and thus to identify a potential trajectory of dissemination of infectious disease into and across the United States. The goal of this review and analysis is to explore the overall effect international adoption and international refugee cases have on the accidental importation of infectious diseases into the United States. The primary objective was to compile a list of some of the more important infectious diseases known to be introduced through international adoptees and refugees. We also provide a comparison of these two populations with particular emphasis on age and country of origin, using data available through the end of the past decade in 2010.

Internet and literature sources were used to compile a significant pool of data from which general trends were identified. Although treatment is not always sufficiently provided or documented before departure for adopted children or refugees, physical exams and health assessments performed after arrival provide useful data for determining prevalence of specific diseases within these incoming populations. For example, “all refugees to the United States are encouraged to obtain a health assessment at local public health departments within ninety days of arrival” [10]. The documentation of such visits provides a substantial basis from which to gauge accounts of old, latent, active, or previous exposure to infectious diseases.

Compilations of data taken from international adoptees often deal with smaller sample sizes than those for refugees but were still viewed as informative. Specific case studies involving few or even one patient are relevant, as such children could serve as the primary source for a foreign infectious disease within US borders. Such studies serve as indicators of potential emerging infectious diseases.

Together, the compiled series of epidemiological evidence allowed for a limited but representative perspective on the potential risk these two specific populations endure under common circumstances. From this, comparisons were made between the two groups to assess the relative degrees of risk and occurrence of infectious disease.

## 2. Basic Assumptions

A staggering 700 million annual human migrations affect residents of recipient localities on both a macro and micro level [10]. Globalization has certainly contributed to economic growth and diversification of populations, but perhaps more importantly, it has also opened up numerous avenues for infectious diseases to be transported along with international adoptees, immigrants, refugees, and tourists.

While it may be expected that immigrants and tourists should have access to basic health care, the environments from which most adoptees and refugees come tend to be substandard, and these populations usually stay in the United States for extended periods of time. Orphanages in embattled countries struggling with political unrest and lack of governmental health programs suffer from inferior standards for sanitation, protection, immunization practices, and preventative treatment. For these reasons, this study focuses on the rise of infectious disease due to international adoptees and refugees. In this brief review, it was a basic assumption that exposures to both adoptee and refugee populations serve as sources of transmission for infectious diseases, which potentially leads to an increase in endemic levels of infectious disease in the new home localities. Of the two populations, international refugees were assumed to present a higher risk due to the higher population numbers and the greater chance of inferior health care once displaced into camps. It might be argued that due to lack of extensive study there is a paucity of direct evidence to support these assumptions. In fact, considering the numbers of individuals arriving from locations with greater risks and exposure to infections of low prevalence in the USA, there is only limited evidence of transmission to the host population. Postarrival transmission, when it occurs, frequently may be more common among the foreign-born population [11, 12] but does occur in the receiving population as well [12, 13]. It is important to note that the few studies that have been done have focused on directly communicable diseases, with little information on the more complicated transmission of vector-borne or other indirectly transmitted diseases.

## 3. Are Infectious Diseases Being Brought into the US?

Several infectious diseases are transmitted by both international adoptees and refugees relocating to the US. Some of these include hepatitis A, hepatitis B, measles, SARS, tuberculosis, syphilis,* Helicobacter pylori*, bacterial gastroenteritis, various intestinal parasites, malaria, and arthropod ectoparasites such as scabies mites and lice. The Yale International Adoption Clinic Experience data demonstrate that adoptees “are at risk for infections well known to be transmitted efficiently within institutional settings” [11]. It would only follow by reason that these same diseases would be efficiently transmitted from infected immigrants within US borders.

The number of international adoptions into the US increased from 1996 through 2010, but the most common countries of origin have remained somewhat stable. The leading country has been China, followed by Russia, Guatemala, South Korea, and Kazakhstan [11]. Thus, infectious diseases endemic in those countries are the most obvious subjects for surveillance and examination.

Hepatitis A has been diagnosed and transmitted within international adoptees and their caretakers. Although often unrecognized because of the exposure's dependency on age, a 2007 report illustrates 5 cases (19%) in adoptees, 2 (7%) in unvaccinated travelers, 13 (48%) in nontraveling contacts of adoptees, and 7 (26%) in contacts of nontraveling contacts of adoptees [12]. Fischer et al. explain this high transmission rate to nontraveling contacts of international adoptees as being a result of asymptomatic hepatitis A present in the children brought into the home of their adoptive parents [12]. Another study, published in 2008, focusing on adopted children from South Korea, identified hepatitis A as a threat to both South Korean children and their contacts within the United States [13]. In 2004, 10 cases were identified in South Korean children of 0 to 9 years of age. Furthermore, 21 cases were confirmed in 2005, and 29 cases were seen in 2006 [13]. This rise in the prevalence of hepatitis A is a cause for concern, as it could potentially impact the epidemiology within the US population with the rising influx of international adoptions.

Hepatitis B has been identified in both internationally adopted children and refugees newly residing in the US. In a study on internationally adopted children, a range of 2% to 5.9% of children from various countries tested positive for active hepatitis B infection, while serological evidence, indicating previous infection or exposure, ranged from 22% to 53% [11]. More specifically, adoptees from South Korea have been identified as having serological markers for hepatitis B [13], and 9% of 164 children adopted from China tested positive for hepatitis B surface antigen, with 28% positive for hepatitis B antibodies [1]. Parents who bring such infected children home are potentially putting themselves and other family members at risk of exposure, and cases of household transmission from such events within the United States have been documented. One report specifically described such hepatitis B transmission from Asian children to their adoptive US families [2].

There also exists significant evidence of international refugees with serologic markers for hepatitis B. Among 12,505 refugees participating in a health study from 1998 to 2001, 70.6% showed active or previous exposure to hepatitis B virus [8]. Also of interest was the fact that 70% of infected refugees were under 30 years of age, and African refugees were 3 times as likely and Asians 2.4 times as likely to be infected compared to Europeans [8].

Hepatitis C infection has also been identified in children from South Korea, but there is much less evidence surrounding the transmission and prevalence. Of concern, however, is the fact that, like most other common diseases in South Korean children, the number of hepatitis C cases increased between 2004 and 2006 [13]. More recent data are needed to determine the risks and transmission status associated with hepatitis C.

Parasitic and other infectious diseases, such as malaria,* Pneumocystis jiroveci* pneumonia, tungiasis, and leprosy, are also sometimes seen in internationally adopted children [14]. There is also strong evidence to suggest that malaria is entering into the United States through sub-Saharan African refugees, though it is not frequently transmitted naturally in the US due to lower exposure to competent* Anopheles* mosquito vectors. The United States accepts an average of 50,000–70,000 refugees each year, and the percentage of these originating in Africa grew significantly from 9% in 1998 to 39% in 2005 [15]. Studies show that in some cases up to 60% of these refugees have arrived infected with malaria, based on parasitemic blood smears 4 weeks after arrival [15]. This is of great concern mainly because of the severity of the disease and the American population's current lack of immunological resistance to malaria [15]. Symptomatic congenital syphilis, although rarely identified, is of concern in international adoptions, as many of these children are adopted from areas where the prevalence of syphilis within the population is increasing. However, despite this general increase, prevalence levels have remained around 1% or less in international adoptees [11]. This speaks to the success of predeparture screening; however, we should not underestimate the potential opportunity for transmission. Miller and Hendrie report in 2000 details serologic evidence for congenital syphilis in a child adopted from China, the country currently leading in the number of international adoptions [1].

Although not identified in international adoptees, per se,* Balamuthia* amebic encephalitis is of concern as demonstrated by Hispanic Americans in California. Seven cases of amebic encephalitis were identified in California residents as part of the California Encephalitis Project. Almost all of them were fatal, and all patients were immunocompetent and of Hispanic American ethnicity [16]. These data, combined with the US Department of State's 72 visas issued for children adopted from Mexico, make this often undiagnosed disease of interest in the spread of infectious disease. With the US Census Bureau's 2006 census estimating the Hispanic American population to be over 15% as of 2010, the potential influx of* Balamuthia* encephalitis, due to the refugee or the adopted population, could put Hispanic Americans at a heightened risk. However, since* Balamuthia*, like other opportunistic amoebae* Naegleria* and* Acanthamoeba*, is generally acquired from natural environmental sources and is present naturally across the US, concern of spread to new populations is likely to be low, and the infections are likely to remain uncommon.

There is also great concern regarding the reemergence of diseases once almost completely eliminated by the US. Tuberculosis is now making a comeback, especially due to drug-resistant strains of the bacteria, and some studies suggest its reemergence partially through international immigrants. For example, a 2006 report by Stout et al. states that “for the first time since the inception of tuberculosis (TB) surveillance in 1951, foreign-born individuals accounted for most of the active TB cases in the United States” [17]. Internationally adopted children have also been shown to present a risk of tuberculosis introduction. Although the majority of adoptees with evidence of tuberculosis are from Russia and China, tuberculin skin tests are positive in 3–5% of children in most studies, with rates as high as 19% in some cases. In 2007, Varkey et al. reported that refugees are at seven times' higher risk of tuberculosis infection than Americans, and twice as high as other foreign-born persons [9]. Measles is another infectious disease set to make a potential comeback, and outbreaks with endemic transmission in the US and Canada have occurred increasingly even through 2015. In 2001, 14 cases were reported after exposure from the travel and care of an adopted child from China [2]. Pertussis is another infectious disease with potential for reemergence within the United States. Although this infection occurs worldwide, the populations at risk are those who are underimmunized or with low immunity. One report identifies a case of pertussis in an adopted infant from Russia; this child's new family and fellow passengers on the flight back to the United States were exposed [2].

Transmission of* Enterocytozoon bieneusi*, resulting in a form of intestinal microsporidiosis, is an example of an uncommon infection occurring in adopted children. In 2003, several cases were identified in a Thai orphanage, and at the time of the survey, none of the infected children showed any gastrointestinal symptoms [18]. This study, reported by Leelayoova et al. in 2005, is evidence for a problem of asymptomatic children not receiving complete health assessments before departure. The lack of symptoms could allow such children to become major sources of infection once displaced into a new unsuspecting population. This warrants increased surveillance, as various enteric and other microsporidioses are regarded as a newly emerging and understudied infectious disease in many parts of the world [19, 20].

Chagas disease, a parasitic disease also known as American trypanosomiasis, has also been shown to move from endemic countries to developed countries and is now widespread in the United States, especially among recent immigrants from Mexico and Central and South America. Immigrants and refugees seeking safety or political asylum have brought in an estimated 56,028 to 357,205 cases of the etiologic agent for this disease,* Trypanosoma cruzi*, or 8 to 50 cases per 1000 legal immigrants [21]. Although Chagas disease is most commonly transmitted by bloodsucking insects of the triatomine reduviid group (commonly known as kissing bugs or assassin bugs), it is also often contracted through blood transfusion, organ transplants, or congenital infection. It is the latter of these mechanisms that allows* T. cruzi* to proliferate within a developed country where the vector bugs are not common or where human contact with them is limited. This is just another example of a disease that should be identified in newly arriving refugees. Failure to do so only contributes to the spread of this disease into nonendemic areas, especially since the disease, like many others, can be zoonotic in wildlife.

Intestinal parasites, both helminthic (worms) and protistan (eukaryotic microbes), also constitute a significant number of infections within both internationally adopted children and refugees. Although the rate of prevalence varies between areas of origin, both populations were found to have intestinal protists or helminths within approximately 9% of the population. The most prevalent parasite in both populations was* Giardia lamblia*, though various other parasites were introduced depending on the country of origin. Reports detailed parasites in the stools in a variety of refugee populations, with their prevalence ranging from 9 to 19%; these studies also identified a substantial number of individuals with more than one parasite or helminth [9, 22]. Another common theme within refugee reports is an increase in frequency of intestinal parasites in children under 18 years of age [22]. In a 2003 review, Chen identified intestinal parasites in international adoptees with a prevalence of up to 51%, again varying with country of origin [2]. The highest prevalence rates occurred in children from Romania, Bulgaria, Moldova, Russia, and China [2]. However, the prevalence of intestinal parasites within internationally adopted children averaged approximately 10% in most studies, though China and Guatemala show a relatively low prevalence rate with only 7% and 8%, respectively [1, 23].

If this importation of* Giardia* does not remain in check, the endemic level could be heightened within the United States borders as illustrated in a 2005 analysis by Ekdahl and Andersson, which demonstrated the change in the epidemiology of giardiasis within Sweden. Data taken from the Swedish national database confirmed that 4,151 cases of* Giardia* in newly arrived immigrants and refugees and 455 cases in internationally adopted children were imported and thus disseminated within the country's borders [24]. This substantial transfer of* Giardia* resulted in a substantially greater calculated risk for being diagnosed with giardiasis. According to Ekdahl and Andersson, “in comparable countries, the calculated risk for being notified with giardiasis was 3–30 times higher in immigrant and refugees than in tourists and 2–5 times higher in adopted children than in immigrants and refugees” [24]. Having identified the potential risk of this known parasite as it crosses US borders, it is advisable to call attention to the need to develop public health measures against travelers and immigrants with this disease.

**Figure 1:**
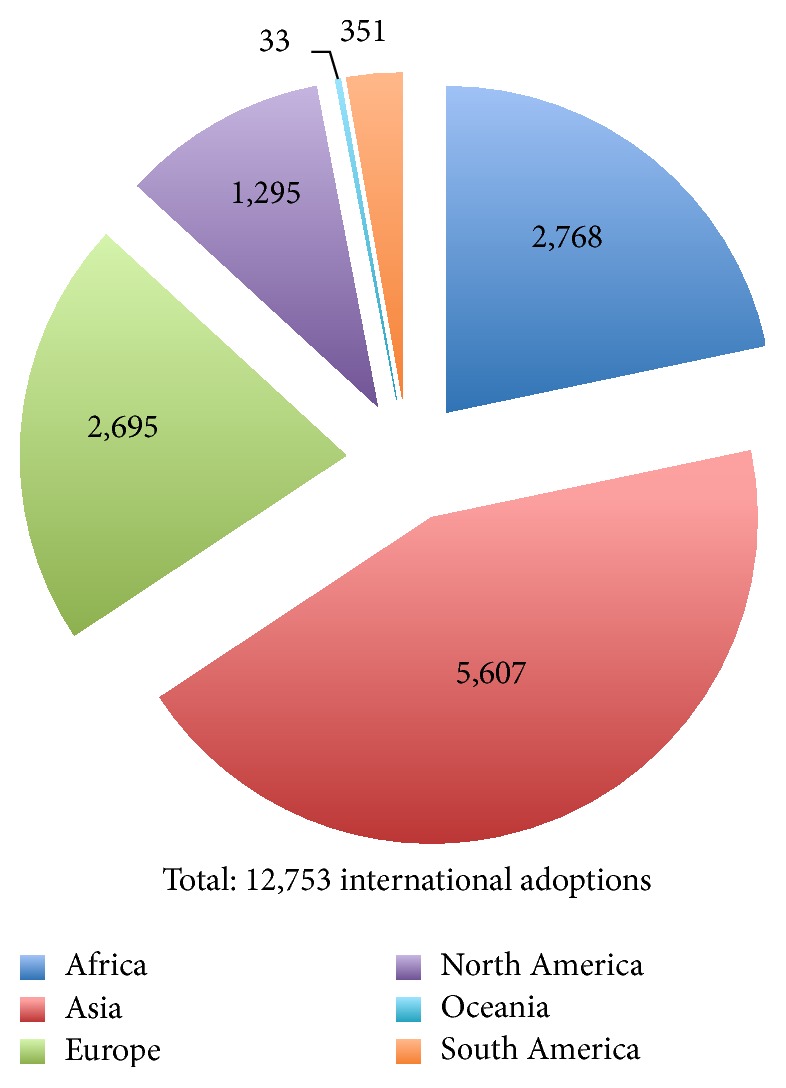
United States international adoption visas issued in 2009 by area of origin. Graphic derived from data provided by US Department of State [25].

**Figure 2:**
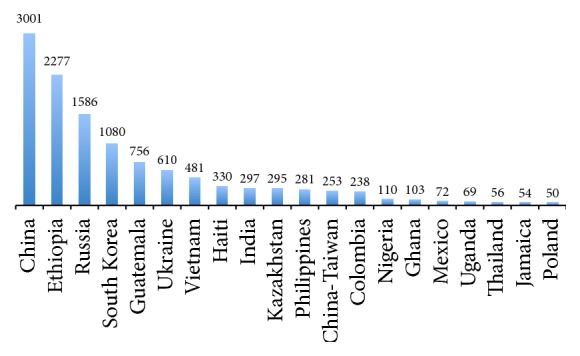
Top 20 most common countries of origin for international adoptees in 2009. Graphic derived from data provided by US Department of State [25].

**Figure 3:**
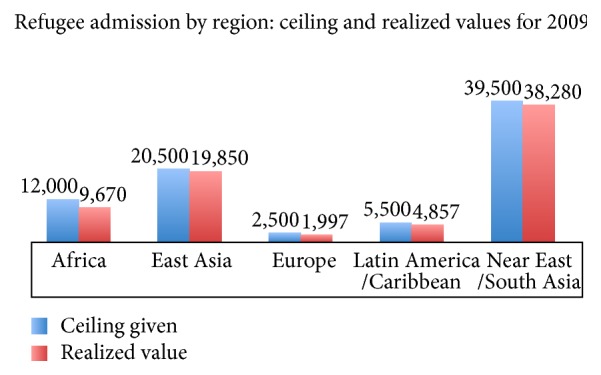
Allotted and realized values of refugees entering the United States by region. Graphic derived from data provided by US Department of State [26].

Based on data from the US State Department, Figure 1 shows the geographic distribution of the 12,753 international adoption visas issued by the United States Department of State in 2009 [25].  Figure 2 illustrates the top twenty countries of origin for international adoptees. These data hold great significance for public health officials as they demonstrate the variety of potential epidemiological backgrounds of internationally adopted children. The United States Department of State allocates refugee resettlements into the United States based upon global region. These regions are divided as Africa, East Asia, Europe and Central Asia, Latin America/Caribbean, and Near East/South Asia. Each year, the US Department of State publishes the proposed ceiling for refugee admissions by region for the coming fiscal year and the realized values for the previous one. The breakdown for 2009 is shown in Figure 3. These data are essential to the study of the globalization of infectious diseases. A total of 74,654 refugees entered the United States in 2009, all of them from areas with diverse epidemiological fingerprints [26]. Having illustrated the presence and transmission of various infectious diseases within these refugee populations, we recommend increasing vigilance and surveillance among large immigrations from various regions of the world, as these are points of considerable concern in the fight against the spread of infectious disease.

## 4. Conclusions

This compilation of data shows that both internationally adopted children and international refugees pose significant potential for infectious disease to be brought into the United States. The infectious diseases commonly transmitted between these two populations are very similar, but the sheer difference in the number of international refugees in comparison to foreign-born adopted children makes the most prevalent diseases of the refugees a greater concern. The poor conditions and frequent lack of medical treatment also contribute to the potential for these diseases to persist and spread among these populations, as they often live in close confinement, and therefore allow greater opportunity for transmission. Families providing homes for international adoptees often have greater access to health care and the means to receive medical attention more quickly than their new wards had in the locality of origin. Nevertheless, each member of these two groups of immigrants has the potential to serve as a source of importation of infectious disease.

The origin of international refugees and internationally adopted children often reflects the political climate of the region as well as the country's social and economic conditions [27]. Despite mandates that children up for adoption must be subjected to predeparture health screening, Hostetter et al. demonstrated in 1989 that 54% of the adopted children they screened once arriving to the United States had an undiagnosed infectious disease at the time of visiting a practitioner; 63% were diagnosed with an unsuspected medical problem regardless of country of origin [28]. Since their study was published, the number of international adoptions and the number of international refugees given admission into the United States have increased, and unfortunately, the data presented in this review do not demonstrate that predeparture detection methods of infectious disease have improved proportionally. However, it is also important to note that not all refugees arrive from refugee camps, and, for those that do, frequently camp medical services, access to care, and predeparture treatment may exceed the levels of local health services in their country of origin. This is clearly the case for many refugees bound for the USA, where CDC-directed treatment programs screen and treat several infections and infestations of public health importance prior to departure.

Finally, it is important to note that zoonotic and other parasitic diseases are frequently emerging in human populations in various parts of the world from which adoptees and refugees regularly migrate to the United States and other countries where those parasites have not yet been recorded but possess characteristics that would allow their colonization of these new localities [29]. An example is the current rapid emergence and spread of canine-associated dirofilariasis caused by* Dirofilaria repens*, throughout Eurasia, and especially in Eastern Europe [30, 31]. The* Aedes* mosquito vectors of this disease and diverse canid reservoirs are present in areas of the United States to which adoptees and refugees might move. The same mosquitoes, currently within the US, are also able to transmit such viral pathogens as dengue, Chikungunya, Zika, and yellow fever, so that health officials as well as entomologists and vector biologists in both the origination and recipient countries should be alert to the possibility of such dissemination.
